# Portable and sensitive approach for hydrazine detection via silver nanoparticles formation: A novel approach for chemical sensing

**DOI:** 10.1016/j.mex.2025.103420

**Published:** 2025-06-06

**Authors:** Sarzamin Khan, Zaibi Zaibi, Muhammad Nasimullah Qureshi, Carlos A.T. Toloza, Eman Alzahrani, Syed Sheraz Ahmad, Leonardo S.G. Teixeira

**Affiliations:** a Department of Chemistry, University of Swabi, Khyber Pakhtunkhwa, Anbar, 23561, Pakistan; b Department of Natural and Exact Science, Universidad de la Costa, Barranquilla, Colombia; c Department of Chemistry, College of Science, Taif University, PO Box 11099, Taif, 21944, Saudi Arabia; d Department of Physics, University of Swabi, Khyber Pakhtunkhwa, Anbar, 23561, Pakistan; e Universidade Federal da Bahia, Instituto de Química, Departamento de Química Analítica, Campus Universitário de Ondina, 40170-115, Salvador, Bahia, Brazil

**Keywords:** AgNPs, Hydrazine, Surface plasmon resonance, Smartphone, Portable and Sensitive Approach for Hydrazine Detection via Silver Nanoparticles Formation: A Novel Approach for Chemical Sensing

## Abstract

A novel silver nanoparticle (AgNPs) based sensor for hydrazine detection is introduced, offering enhanced sensitivity, selectivity and a swift response time. The sensing mechanism relies on the reduction of silver ions in the presence of analyte (hydrazine), leading to the formation of silver nanoparticles under optimized conditions, producing a distinct and intense surface plasmon resonance signal. Moreover, the distinct color variations have been quantitatively assessed through RGB (red, green, and blue, primary component of light) values by smartphone, allowing its functionality as a portable hydrazine sensor. The proposed sensing strategy is highly sensitive, does not rely on complex equipment, and is applicable for field based hydrazine analysis in industrial and natural water systems.

• Indirect colorimetric detection of hydrazine using in situ synthesize silver nanoparticles
• Integration with smartphone enables portable, on-site detection of hydrazine
• Enhanced selectivity and sensitivity, make the sensor ideal for hydrazine monitoring in environmental samples

## Graphical abstract

**Figure:**
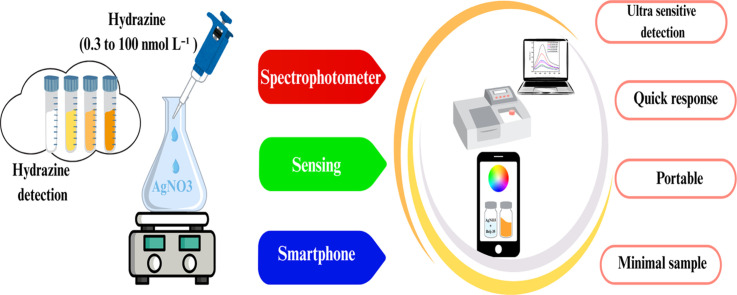

Specifications table

**Table:** 

Subject area	Chemistry
More specific subject area	Analytical chemistry
Name of method	Portable and Sensitive Approach for Hydrazine Detection via Silver Nanoparticles Formation: A Novel Approach for Chemical Sensing
Name and reference of original method	A smartphone assisted colorimetric sensing platform for the detection of hydrazine at ultra-trace levels in water samples based on the formation of silver nanoparticles

## Background

**Scheme 1:**
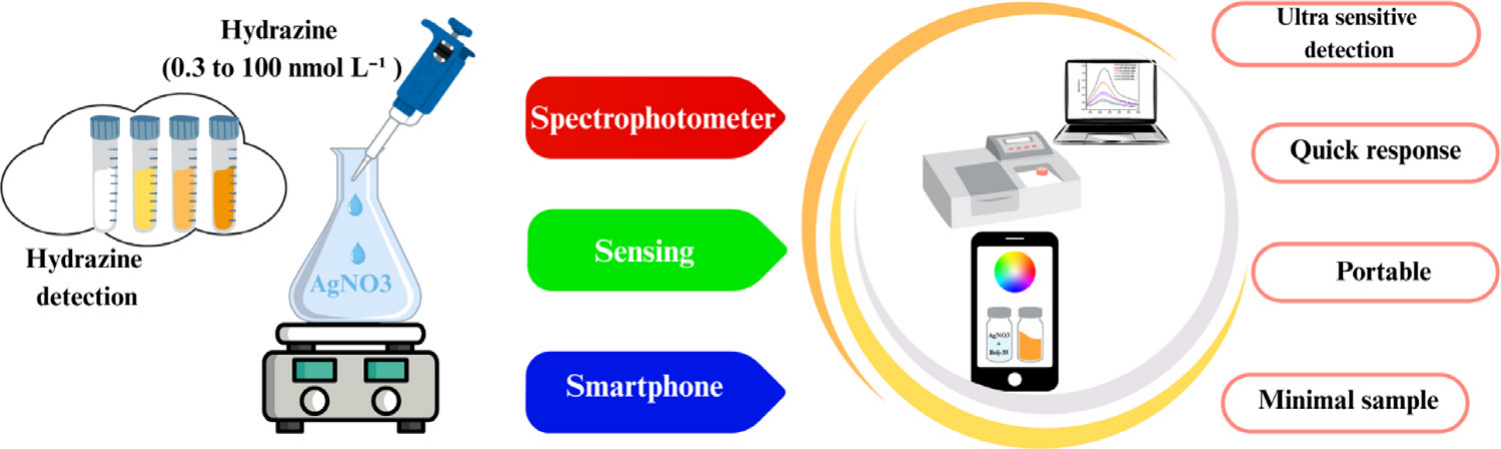
Schematic representation of hydrazine detection based on formation of silver nanoparticles.

Hydrazine (N₂H₄) is a highly toxic and carcinogenic compound, known for its significant environmental and health hazards. It causes notable damage to DNA, kidneys, and the brain, and is associated with hematological and respiratory disorders [1]. It is additionally classified as both a neurotoxin and hepatotoxin [2,3]. Beyond its initial application as a rocket propellant, it has been utilized as a key reagent in various sectors, including the chemical, pharmaceutical, agricultural, polymer, and textile industries. Therefore, the development of sensitive, selective, and portable methods for its detection is of considerable importance in environmental monitoring and public safety. A wide array of analytical methods has been established for the detection of hydrazine in various sample matrices [4]. However, due to the lack of inherent chromophoric groups in hydrazine, direct detection via fluorescence or UV–visible absorbance is not feasible without prior chemical derivatization, that are complex and time-consuming. Recent advances in nanotechnology have paved the way for nanoparticle-based colorimetric sensors, which offer excellent sensitivity, selectivity, and visual signal output without the need for complex instruments. Among these, silver nanoparticles (AgNPs) have attracted attention due to their unique optical properties and strong response to chemical changes in their surrounding environment. In this context, a novel method has been developed for the sensitive and selective detection of hydrazine using a silver nanoparticle-based sensing system. The detection strategy leverages the reducing capability of hydrazine, which facilitates the *in-situ* formation of AgNPs from silver ions. This reaction induces a visible color change that can be quantitatively analyzed through smartphone-assisted RGB analysis, enabling portable and low-cost sensing. Conventional smartphone-based color detection methods reply on pre-synthesized nanoparticles or organic dyes, that alter color upon interaction with the analyte. In the proposed protocol, hydrazine itself reduces silver ions to form AgNPs, eliminating the need for separate nanoparticles synthesis. Furthermore, hydrazine selectively reduces Ag^+^ to Ag^0^, significantly suppressing the interferences from matrix. The response is quick (<5 min), with visible color changes, enabling direct application to environmental samples without complex pretreatment. Under optimized conditions (2.5 × 10⁻⁴ mol L⁻¹ silver ion concentration, pH 7.5, Brij-35 as a stabilizer, and a reaction time of 4.0 min) the method achieves a broad spectrophotometric detection range (0.3–100 nmol L⁻¹). Additionally, the smartphone-based protocol enables nanomolar level hydrazine detection (15.0–45.0 nmol L⁻¹). In literature several methods have been reported for hydrazine detection, for example Zargar *et.al.,* reported a colorimetric sensor in which hydrazine reduced AuCl^4-^ to AuNPs, enabling spectrophotometric detection with a linear range of 6.0 to 40.0 µ molL^-1^ and limit of detection of 1.1 µ molL^-1^. However, the method is less sensitive, relatively expensive, and lacks smartphone-based detection, limiting its applicability for portable detection [5]. Prussian blue nanoparticles (PBNPs) have also been explored for hydrazine detection. In this approach, hydrazine reduces Fe³⁺ to Fe²⁺, turning PBNPs from blue to colorless. This method achieved an LOD of 0.2 μM (spectrophotometer) and 9 μM (portable device). Despite its advantages, the assay is less sensitive an may suffer from interference by strong reducing agents in the sample that can also reduce Fe³⁺ to Fe²⁺ in PBNPs [6] (Scheme 1).

## Method details

In this paper we report a new established procedure recently introduced for the sensitive and selective determination of hydrazine [7]. The study presents a highly sensitive AgNPs based sensing platform for hydrazine detection. The sensing mechanism relies on the direct reduction of silver ions by hydrazine, enabling the *in-situ* formation of AgNPs. Under precisely optimized experimental conditions wherein the silver ion concentration is maintained at 2.5 × 10⁻⁴ mol L⁻¹, keeping the pH at 7.5, and Brij-35 is employed as the stabilizer with the reaction time of 4.0 min, exhibits a dynamic response across a broad analytical range spanning from 0.3 to 100 nmol L⁻¹ hydrazine. Additionally, smartphone-assisted RGB color analysis has been employed to interpret visual color changes, making this approach suitable for portable sensing of hydrazine at a nanomolar concentration (15.0 nmol L⁻¹ to 50.0 nmol L⁻¹).

### Instruments and apparatus

The weighing of samples was conducted using an OHAUS analytical balance (model SPX2202), with sensitivity of 10⁻³ g and an accuracy of ± 0.01 mg. A digital pH meter (ADWA, AD1020, USA) was utilized for precise estimation of the hydrogen ion concentration in different solutions. UV–Vis spectra were acquired using double beam spectrophotometer (SHIMADZU UV-1800) equipped with 1.0 cm path length quartz cuvettes. For portable sensing a smartphone-based system was adapted (Model: Samsung Galaxy A15), placed at 15.0 cm distance from sample vials. Micrographs of nanoparticles were obtained by TEM (JEOL 2010 Instrument), operating at 200 kV.

### Chemicals and reagent

The chemical reagents like the hydrazine monohydrate (N_2_H_4_.H_2_O, 98.0 %), silver nitrate (99.9 %), hetaethylene glycol monododecyl ether (90 %), cetrimonium bromide (CTAB, &gt;98 %), sodium lauryl sulfate (SLS, &gt;99.5 %), were acquired from Sigma Aldrich. All solutions were prepared using deionized water possessing a resistivity of ∼18 MΩ·cm. A range of metal salts used in interference studies included sodium chloride, sodium cyanide, sodium nitrate, magnesium sulfate, potassium chloride, calcium chloride, ferric chloride and zinc chloride were also of analytical grade. A standard solution of 2.0 mmol L ^−1^ hydrazine was obtained by dissolving appropriate quantity of hydrazine monohydrate in 5.0 mL of distilled water. A fresh working solution was prepared on daily basis by appropriate dilution.

### Procedure

#### Preparation of standard and sample solution of hydrazine

Stock standard solutions of analyte were prepared at 2.0 mmol l^-1^ by the addition of specified amount of hydrazine in ultrapure water. While dilution of the stock solutions with water was performed to prepare less concentrated working solutions. The environmental aqueous samples were filtered through 0.45 µm filter paper and were analyzed for hydrazine detection without further treatment.

#### Synthesis of silver nanoparticles

**Fig. 1:**
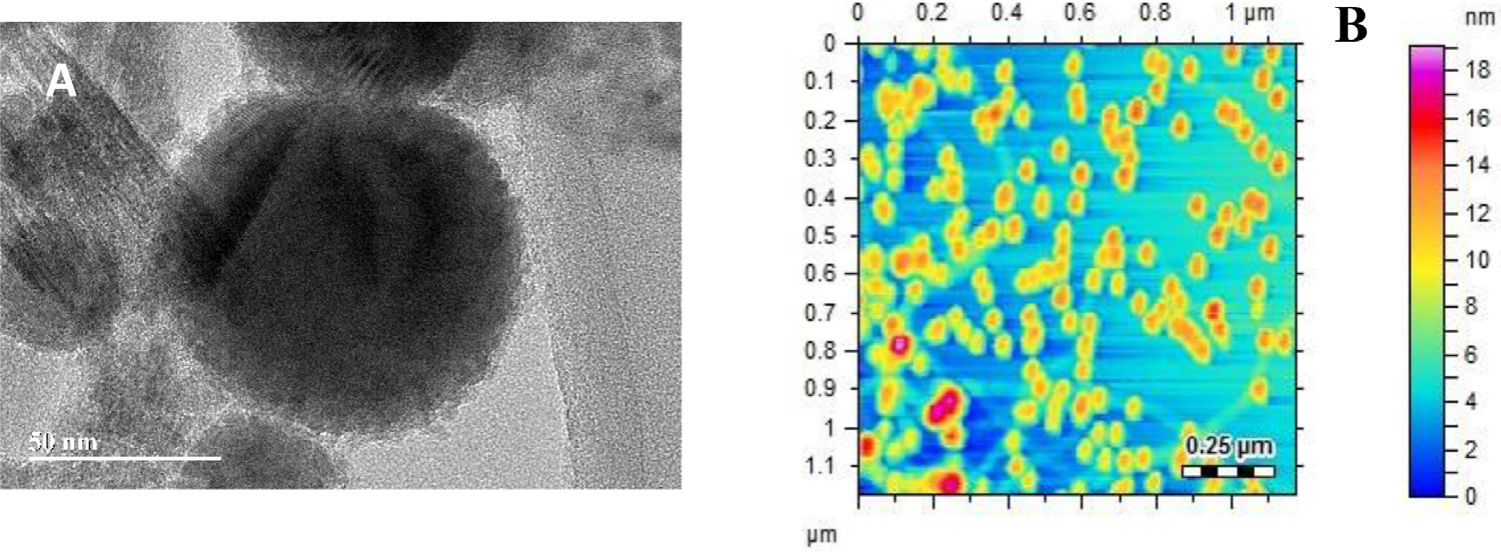
Characterization of synthesized AgNPs(A) TEM and (B) AFM.

In a standard hydrazine detection protocol, different amount of hydrazine was introduced into a 5.0 mL of freshly prepared 0.25 mmol L⁻¹ AgNO₃ solution. Following this the reaction mixture was then subjected to stirring after adjusting the pH to 7.5 (final volume: 40.0 mL). Within a few seconds, the appearance of a pale-yellow color signified the formation of AgNPs. After 4.0 min, an aliquot of the reaction solution was carefully transferred into a quartz cell, and its absorbance was recorded at 420 nm (surface plasmon resonance peak) [8]. The HTREM and AFM micrographs confirmed the formation of AgNPs as shown in Fig. 1.

#### Order of reagent addition

The optimized order of mixing of reagent was the addition of stabilizer to silver ions followed by pH adjustment, and finally interaction with hydrazine. The order was found critical for ensuring the controlled formation of AgNPs, leading to robust and reproducible analytical response.

## Method validation

**Fig. 2:**
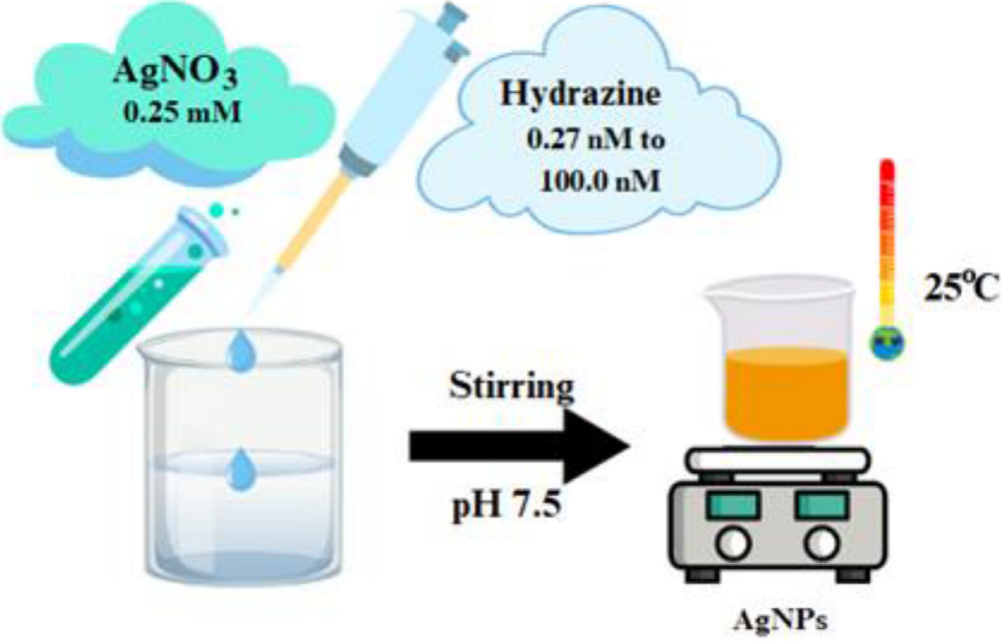
Synthesis of silver nanoparticles and subsequent detection of hydrazine.

**Fig. 3:**
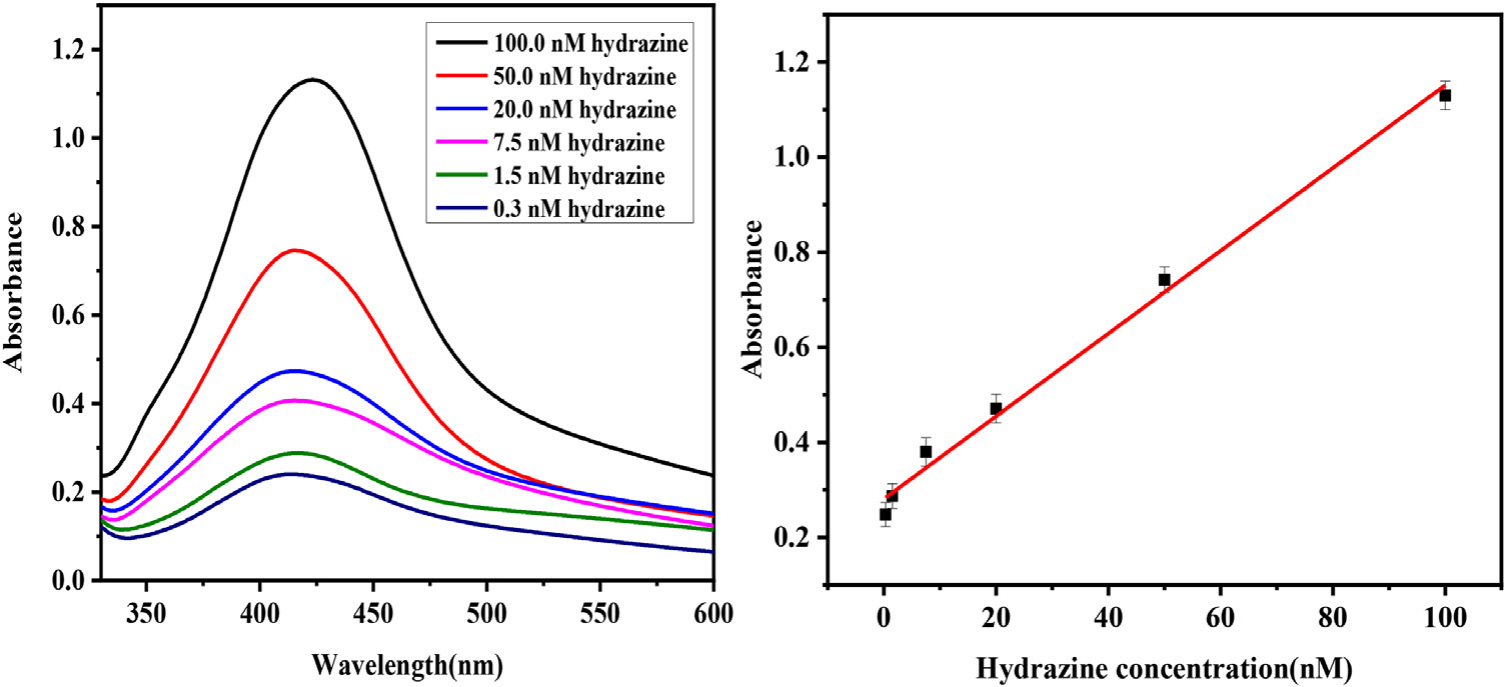
(a) Variations in sensing response with varying hydrazine concentration (0.3 to 100.0 nmol L⁻¹) at optimized experimental parameters (response time: 4 min; Ag⁺: 25 mmol L⁻¹; pH 7.5; Brij-35). (b) Linear response of the sensor between the absorbance and concentration of hydrazine upto 100.0 nmol L⁻¹. Each calibration points displaying the relative standard deviation for three number of analyses (*n = 3*).

The described analytical protocol employs a plasmonic nanosensor approach for the ultra-trace quantification of hydrazine, exploiting the in-situ reduction of Ag⁺ ions to colloidal silver nanoparticles (AgNPs) as an optical probe (Fig. 2). The reaction, occurring in a mildly buffered aqueous media (pH 7.5), results in a distinctive localized surface plasmon resonance (LSPR) absorption peak at 420 nm, indicated the AgNPs formation (Fig. 3). Following 4.0 min of reaction, an aliquot of the reaction mixture was taken and placed into a quartz cuvette for absorbance measurements.

## Linearity and calibration curve

The linearity of the proposed hydrazine detection method was evaluated using both spectrophotometric absorbance measurements and smartphone-assisted RGB color analysis, offering dual analytical approaches for versatile application. A series of hydrazine standard solutions, ranging from 0.3 to 100 nmol L⁻¹, were analyzed under optimized conditions; silver ion concentration of 2.5 × 10⁻⁴ mol L⁻¹, pH 7.5, Brij-35 as a stabilizer, and a reaction time of 4.0 min. In the spectrophotometric approach, the absorbance corresponding to the surface plasmon resonance (SPR) of *in-situ* formed AgNPs was recorded, yielding a well-defined calibration curve with a strong linear relationship (R² > 0.99) across the full concentration range. Simultaneously for hydrazine quantification, smartphone-based image acquisition was conducted manually. The silver nanoparticle solutions were imaged pre- and post-hydrazine addition using the back camera (50 MP) of a Samsung Galaxy A15. Photographs were taken 5.0 min after the reaction began, inside a light-controlled enclosure to avoid ambient light interference. The interior was lined with black strips to reduce reflections, and a 1.5 m LED strip was installed to ensure consistent illumination. The vial containing the probe solution was placed 15.0 cm in front of the smartphone, this distance was selected based on preliminary tests to achieve optimal focus and image clarity. For consistency, images were captured from a fixed position in every analysis. Each sample was photographed multiple times and the best-focused image was selected for processing.

The acquired images were transferred into a computer and analyzed using ImageJ software to quantify the mean RGB color channel intensities. The calculated absorbance for each channel was plotted against the hydrazine concentration in the sensing platform to find the appropriate channel for the best linear fit. The experimental finding revealed (Table 1) that the green channel provides appropriate linearity (R^2^ = 0.998) in 15.0 to 45.0 nmol l^-1^ concentration range with limit of detection of 4.5 nmol l^-1^. Therefore, the green channel was employed for hydrazine detection in aqueous samples.

**Table 1:** Smartphone based detection of hydrazine.

Hydrazine (nM)	R	G	B
0	0	0	0
15	0.06698	0.09297	0.04592
22.5	0.10922	0.14707	0.06999
30	0.16106	0.21536	0.09597
45	0.24868	0.32816	0.15093

## Selectivity study

**Fig. 4:**
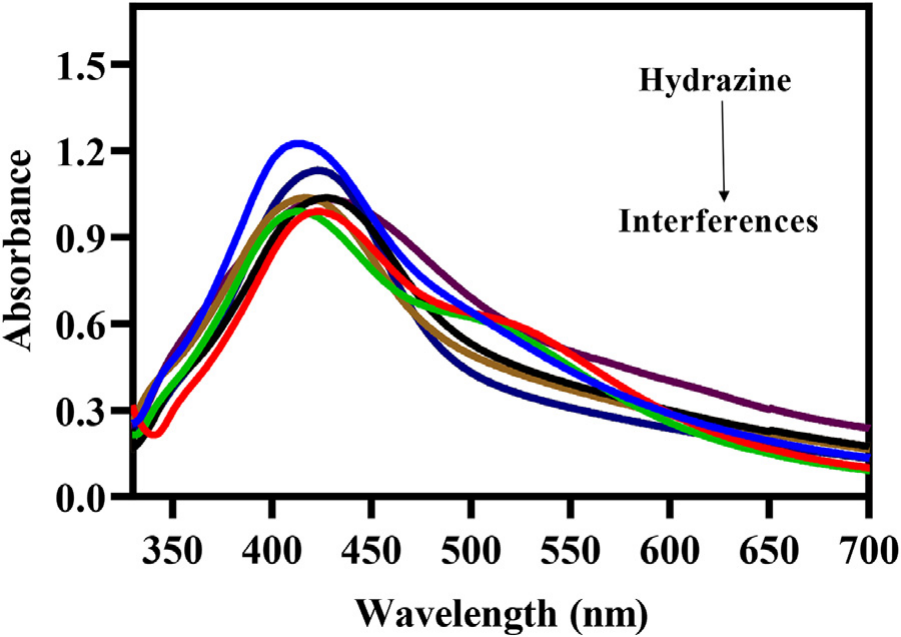
Effect of various metal cations (Na(I), K(I), Ca(II), Fe(III), Mg(II), Zn(I)) and anions (Cl(I), NO_3_(I), SO_4_(II), PO_4_(III), HCO_3_(I) and SO_3_(II)) on hydrazine detection.

The effectiveness of the hydrazine method was assessed in real water samples. The suggested spectrophotometric method was applied to estimate hydrazine levels in various wastewater samples Fig. 4. Several spiked wastewater samples were prepared by adding hydrazine ions. The main components of this wastewater were: Na(I), K(I), Ca(II), Fe(III), Mg(II), Zn(I) and many anions such as: Cl(I), NO_3_(I), SO_4_(II), PO_4_(III), HCO_3_(I) and SO_3_(II). The suggested new procedure has also been utilized for the hydrazine determination in a boiler water sample. The hydrazine concentrations determined by the proposed spectrophotometric method were consistent with those obtained from previously reported methods, demonstrating high accuracy in the presence of various matrices [9]. The use of surfactants (anionic, cationic and non-anionic) significantly improved the sensitivity and selectivity of the method for hydrazine measurement in the ng·mL⁻¹ range.

## Conclusion

Herein, a smartphone based colorimetric protocol is established for pg level detection of hydrazine in environmental samples. The assay relies on hydrazine induced formation of AgNPs formation. The controlled experimental parameters showed increasing absorbance with hydrazine concentration at fixed wavelength. Contrary to the reported methods, this protocol provides a simple and real time alternative for detection of hydrazine at ultra trace levels.

## Limitations

None.

## Ethics statements

This study was carried in strict accordance with the ethical guidelines of the Methods X journal.

## CRediT author statement

**Sarzamin Khan:** Investigation, conceptualization, methodology, writing original draft **Zaibi:** formal analysis, data curation, writing original draft **Muhammad Nasimullah Qureshi:** conceptualization, writing original draft **Syed Shiraz Ahmad:** visualization, investigation **Carlos A. T. Toloza:** formal analysis, investigation **Eman Alzahrani:** Software, formatting, **Leonardo S. G. Teixeira:** formal analysis, resources.

## Funding

This research was funded by Taif University, Saudi Arabia, Project No (TU-DSPP-2024–18).

## Declaration of competing interest

The authors declare that they have no known competing financial interests or personal relationships that could have appeared to influence the work reported in this paper.

## Data Availability

Data will be made available on request.
